# Nucleic acids in inclusion bodies obtained from *E. coli* cells expressing human interferon-gamma

**DOI:** 10.1186/s12934-020-01400-6

**Published:** 2020-07-11

**Authors:** Elena Krachmarova, Ivan Ivanov, Genoveva Nacheva

**Affiliations:** Institute of Molecular Biology “Roumen Tsanev”, Bulgarian Academy of Sciences, Academic Georgi Bonchev Str., Blok 21, 1113 Sofia, Bulgaria

**Keywords:** Inclusion bodies, Nucleic acids, *E. coli*, Recombinant protein production, Human interferon-gamma, Protein aggregation

## Abstract

**Background:**

Inclusion bodies (IBs) are protein aggregates in recombinant bacterial cells containing mainly the target recombinant protein. Although it has been shown that IBs contain functional proteins along with protein aggregates, their direct application as pharmaceuticals is hindered by their heterogeneity and hazardous contaminants with bacterial origin. Therefore, together with the production of soluble species, IBs remain the main source for manufacture of recombinant proteins with medical application. The quality and composition of the IBs affect the refolding yield and further purification of the recombinant protein. The knowledge whether nucleic acids are genuine components or concomitant impurities of the IBs is a prerequisite for the understanding of the IBs formation and for development of optimized protocols for recombinant protein refolding and purification. IBs isolated from *Escherichia coli* overexpressing human interferon-gamma (hIFNγ), a protein with therapeutic application, were used as a model.

**Results:**

IBs were isolated from *E. coli* LE392 cells transformed with a hIFNγ expressing plasmid under standard conditions and further purified by centrifugation on a sucrose cushion, followed by several steps of sonication and washings with non-denaturing concentrations of urea. The efficiency of the purification was estimated by SDS-PAGE gel electrophoresis and parallel microbiological testing for the presence of residual intact bacteria. Phenol/chloroform extraction showed that the highly purified IBs contain both DNA and RNA. The latter were studied by UV spectroscopy and agarose gel electrophoresis combined with enzymatic treatment and hybridization. DNA was observed as a diffuse fraction mainly in the range of 250 to 1000 bp. RNA isolated by TRIzol^®^ also demonstrated a substantial molecular heterogeneity. Hybridization with ^32^P-labelled oligonucleotides showed that the IBs contain rRNA and are enriched of hIFNγ mRNA.

**Conclusions:**

The results presented in this study indicate that the nucleic acids might be intrinsic components rather than co-precipitated impurities in the IBs. We assume that the nucleic acids are active participants in the aggregation of recombinant proteins and formation of the IBs that originate from the transcription and translation machinery of the microbial cell factory. Further studies are needed to ascertain this notion.

## Introduction

*Escherichia coli* is one of the most preferable microbial factory for production of recombinant proteins for research, diagnostics and medical use because of its easy, fast and cheap cultivation, well investigated genetics and physiology as well as for the availability of numerous tools for genetic manipulation [1 and references therein]. It is well known that the overexpression of eukaryotic proteins in *E. coli* often causes formation of inclusion bodies (IBs) [2], containing mainly improperly folded proteins [3]. For a long time, it has been believed that the aggregated proteins are immunogenic and partly or completely devoid of biological activity [4]. At present, increasing evidence show that IBs have amyloid-like structure and comprise aggregated as well as native folded proteins with preserved biological activity [2]. This fact, together with the mechanical stability and high porosity of the IBs has defined them as unconventional functional materials with a wide spectrum of applications in biotechnology and biomedicine [5–7]. Recently, first report towards in vitro preparation of tailored and chemically defined IBs with potential for clinical application was published [8]. In spite of these first steps, the large-scale clinical application of IBs is still hindered by their undefined heterogeneous composition and the presence of hazardous contaminants from the bacterial cell, especially endotoxins [9]. Thus IBs remain a main source for production of pure biologically active recombinant proteins for medical purposes that can be isolated upon cell disruption, solubilisation, subsequent refolding and purification [10]. Furthermore, to turn IBs aggregation into greatest use, new combinatorial approaches at each step was proposed [11].

Protein folding and IBs formation is extensively discussed in a number of reviews [1, 12–15]. For a long time, protein aggregation has been considered as a process driven by hydrophobic interactions between fully-denatured protein molecules, however, increasing evidence indicate that protein aggregates are composed of partially unfolded or misfolded proteins linked by unspecific hydrophobic interactions [14, 16, 17]. Therefore, aggregation seems to be a competitive reaction to folding, depending on specific folding behaviour and conditions [18]. It is influenced by various factors such as protein size, presence of specific hydrophobic compartments in the molecule, pI, protein abundance [19, 20], high local concentration of the polypeptide chains emerging from ribosomes [21], limited amount of bacterial chaperones and proteases, which affect either folding or degradation of the unfolded or misfolded polypeptides [22, 23].

The chemical composition of IBs still remains obscure. It varies in a broad range and depends on the properties of the specific recombinant protein, fermentation conditions, host genetic background, IBs purification procedures, etc. [18]. Apparently, the major component of the bacterial IBs is the target recombinant protein [24–27]. In addition, they can also contain various contaminants such as lipids, nucleic acids, endogenous cell proteins, chaperones, etc. [28]. Since chaperons assist in protein folding, they are the main cell components “controlling” protein aggregation [29]. Among them, the heat shock proteins IbpA and IbpB [30–32] and DnaK and GroEL [32, 33] have been found in bacterial IBs. There is evidence that the IBs contain also plasmid DNA [34], ribosomal RNA, RNA polymerase subunits [35, 36], ribosomal proteins L13 [36], L7 and L12 [24], elongation factor Tu [37], membrane proteins OmpF, OmpC, and OmpA [24], membrane phospholipids and cellular RNAs [38]. Based on these findings some authors assume that the protein aggregation in vivo occurs simultaneously with the protein synthesis [35, 36]. Taking into account that in most of these studies the IBs have not been precisely purified, Rinas and Bailey [24] suggest that the cellular ingredients found in IBs co-precipitate during their isolation.

The quality and composition of the IBs affect the refolding yield and further purification of the recombinant protein. It has been shown that fully denatured mammalian proteins show unusually high solubility in nucleic acid-free pure water [39]. Since the recombinant proteins that are produced as pharmaceuticals are mainly mammalian proteins, the presence of nucleic acids in their preparation is critical for their refolding because the nucleic acids actively participate in the protein aggregation process via direct electrostatic interactions with partially folded or unfolded proteins. The literature survey shows that most of the studies on *E. coli* IBs carried so far are focused on protein composition and mechanisms of aggregation, whereas the data concerning nucleic acids are scarce and vague. Bearing in mind that the recombinant proteins manufactured for medical applications should be free of nucleic acids, we have focussed in this study on the content and nature of nucleic acids in highly purified *E. coli* IBs. As a model in this study we use IBs isolated from *E. coli* LE392 overexpressing human interferon-gamma (hIFNγ).

## Results

### Purification of hIFNγ IBs

**Fig. 1:**
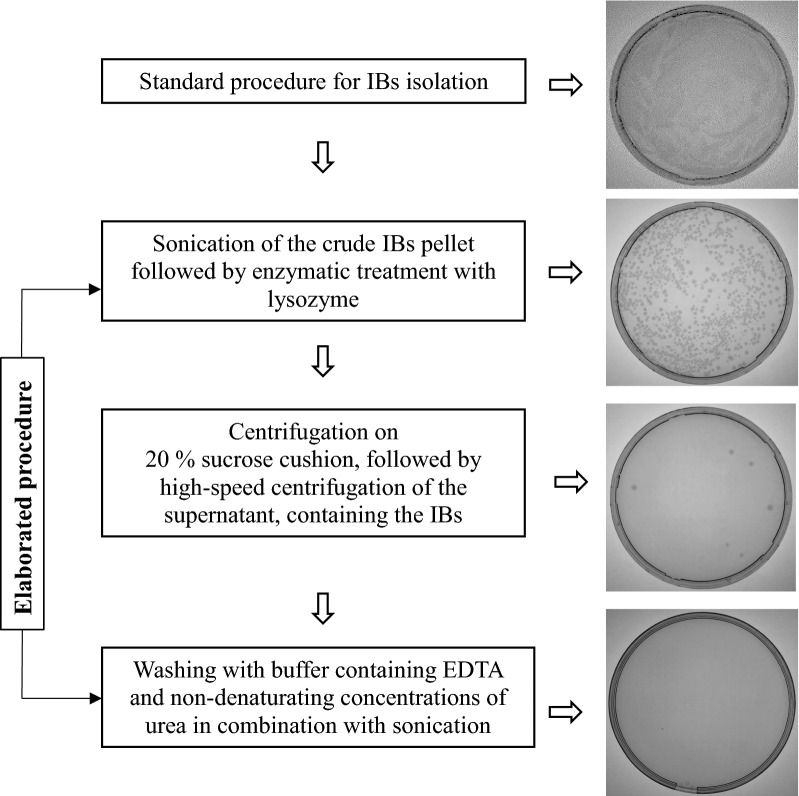
Schematic illustration showing the IBs purification steps and analysis for presence of viable bacterial cells

To study the type of nucleic acids co-aggregating with recombinant hIFNγ in *E. coli* cells we developed a three step procedure for purification of IBs from intact bacterial cells and subcellular components (Fig. 1). The first step included additional sonication of the crude IBs pellet followed by treatment with 100 μg/ml lysozyme. According to literature data, the combination of these two procedures should lead to IBs free from intact bacterial cells [40]. We observed, however, that substantial amount of viable *E. coli* cells still remained in the pellet (Fig. 1). The latter were successfully removed by centrifugation on a cushion of 20% sucrose that comprised the second step of the purification procedure. Further the IBs were collected by centrifugation of the supernatant at 13,000 rpm for 20 min at 4 °C. The pellet thus obtained contained negligible amount (sporadic) of bacterial cells (Fig. 1). During the third purification step the IBs were washed twice with washing buffer containing EDTA and non-denaturing concentrations of urea (1 M). Between the washing steps the IBs were sonicated and collected by centrifugation. This procedure proved to be essential for the quality and yield of the purified IBs since no bacterial growth was further observed (Fig. 1).

**Fig. 2:**
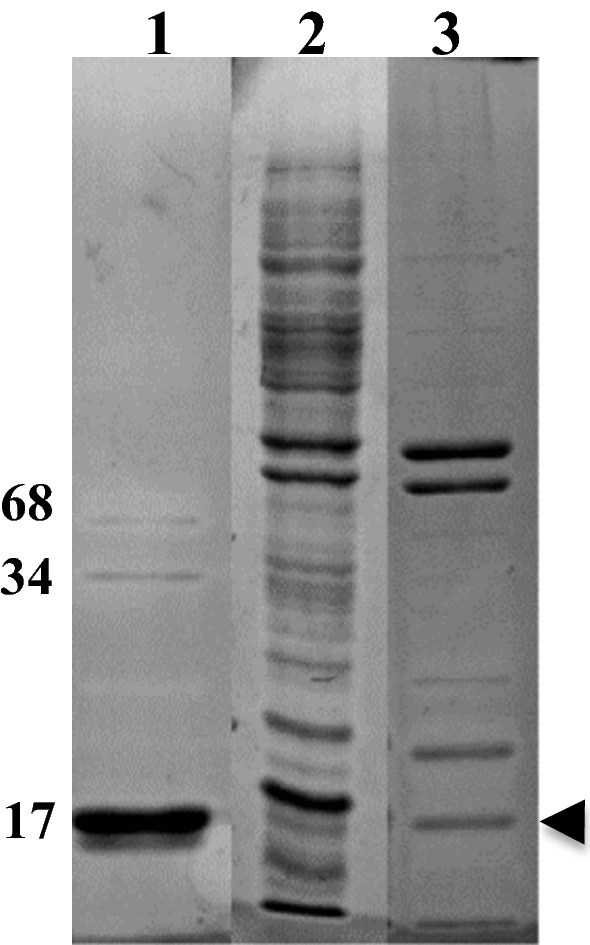
SDS PAGE electrophoretic pattern of hIFNγ IBs. 1—Purified hIFNγ obtained as described in [41] was used as a standard, the molecular weight of the monomer and the covalently bound dimer and tetramer are shown in kDa; 2—Crude IBs; 3—IBs after the last purification step 3

The IBs electrophoretic pattern was also used as a criterion for their purity. Figure 2 illustrates the significant difference in the protein composition of the IBs prepared by the standard procedure [41] and after the additional purification.

### Characteristics of nucleic acid in purified hIFNγ IBs

Nucleic acids were isolated from purified IBs by phenol–chloroform extraction and precipitation with ethanol. Their concentration and UV spectra were analysed by NanoDrop^®^. The UV spectra showed absorption maxima at 260 nm and typical for the nucleic acids A_260/280_ and A_260/230_ ratios (Additional file 1: Fig. S1). The agarose electrophoretic pattern of the isolated total nucleic acids (Fig. 2a) revealed two main fractions—a high-molecular weight fraction (above 10,000 bp) and a more diffusive one in the range of 250 to 1000 bp.

**Fig. 3:**
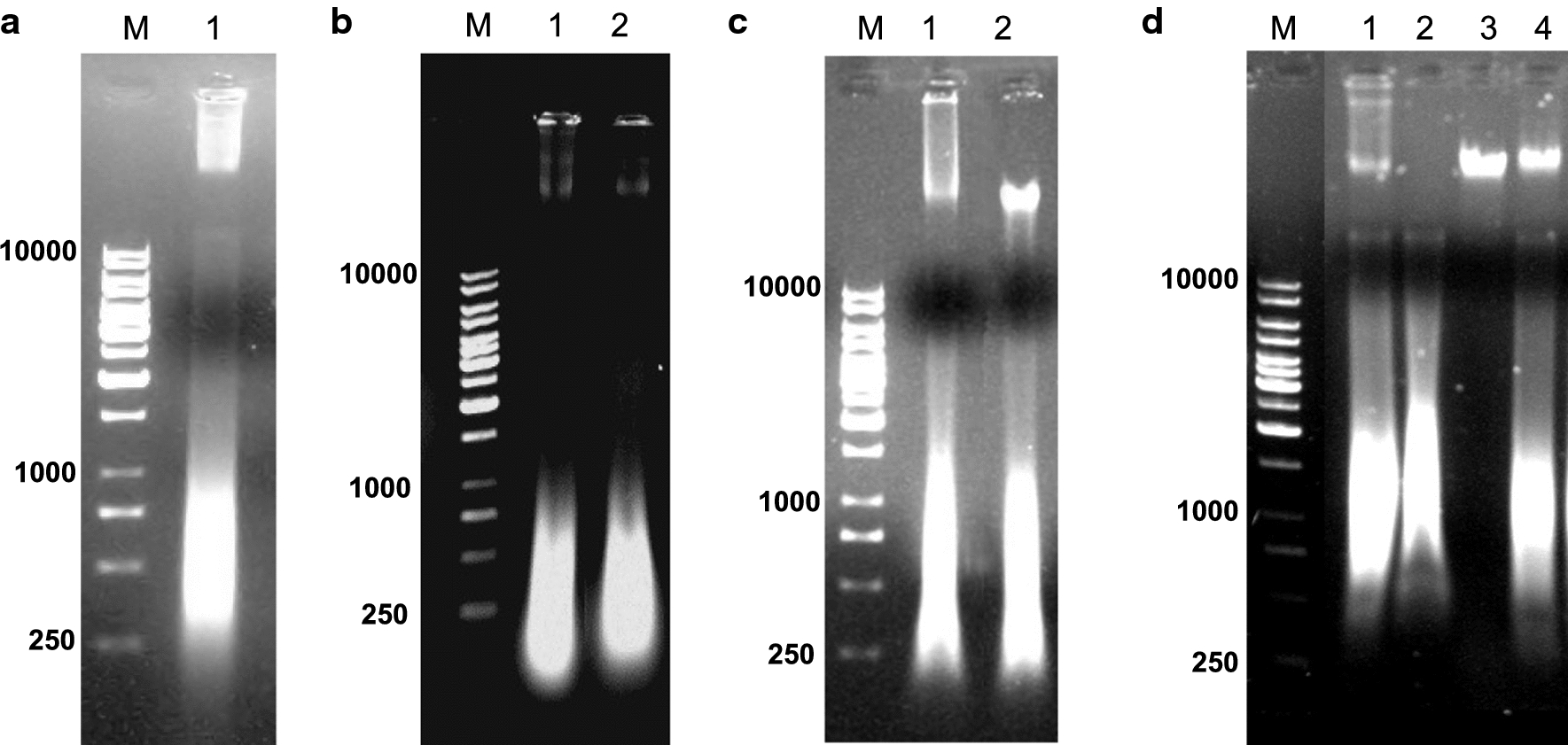
**a** Agarose gel-electrophoresis of nucleic acids isolated from purified hIFNγ IBs. *1*—Sample, isolated from IBs, **b** Agarose gel-electrophoresis of nucleic acids isolated from purified hIFNγ IBs treated with restriction endonuclease XhoI and **c** HindIII. *1*—Non-treated sample; *2(b)*—Sample treated with XhoI; *2(c)*—sample treated with HindIII; **d** Enzymatic digestion of nucleic acids isolated from purified hIFNγ IBs *1*—non treated sample; *2*—sample treated with mixture of RNase A and RNase T_1_; *3*—sample treated with DNase I; *4*—sample treated with Proteinase K; *M*—Molecular weight marker, bp

One can speculate that the low mobility fraction (conditionally called high-molecular, Fig. 3a) consists of genomic or multimeric plasmid DNA, or a mixture of both. In order to investigate the origin of this fraction, samples were treated with the restriction endonucleases XhoI and HindIII. They were chosen because of the fact that the *E. coli* genome has multiple restriction sites for both enzymes, while no XhoI site exists in the expression plasmid pP1SD-hIFNγ. Therefore, any change in the mobility of the high-molecular fraction upon treatment with XhoI would mean that it consists of genomic DNA. Since the expression plasmid bears a unique HindIII site, the treatment with this enzyme would generate a distinguished single fraction from eventual plasmid concatemers.

As seen from Fig. 3b, c, the digestion with neither of the enzymes resulted in any significant change in the mobility of the high-molecular fraction. Its resistance to both restriction endonucleases suggested that this fraction might be composed of RNA rather than DNA. In order to check this assumption, samples were further treated with DNase I and a mixture of RNase A and RNase T1. In addition, to test for the presence of residual proteins that could form stable complexes with the nucleic acids, the sample was also treated with Proteinase K.

Figure 3d shows that the mixture of RNase A and RNase T_1_ resulted in a complete hydrolysis of the high-molecular fraction thus confirming that it consists of RNA fragments that under non-denatured electrophoresis conditions have preserved higher order structures and migrate slow. Surprisingly, DNase I degraded entirely the high mobility fraction (250–1000 bp). This means that it is composed of very heterogeneous in size DNA fragments. Proteinase K did not cause changes in the mobility of any electrophoretic fraction thus proving that the nucleic acids isolated from IBs were devoid of interfering proteins.

### Characterization of RNA isolated from purified hIFNγ IBs

**Fig. 4:**
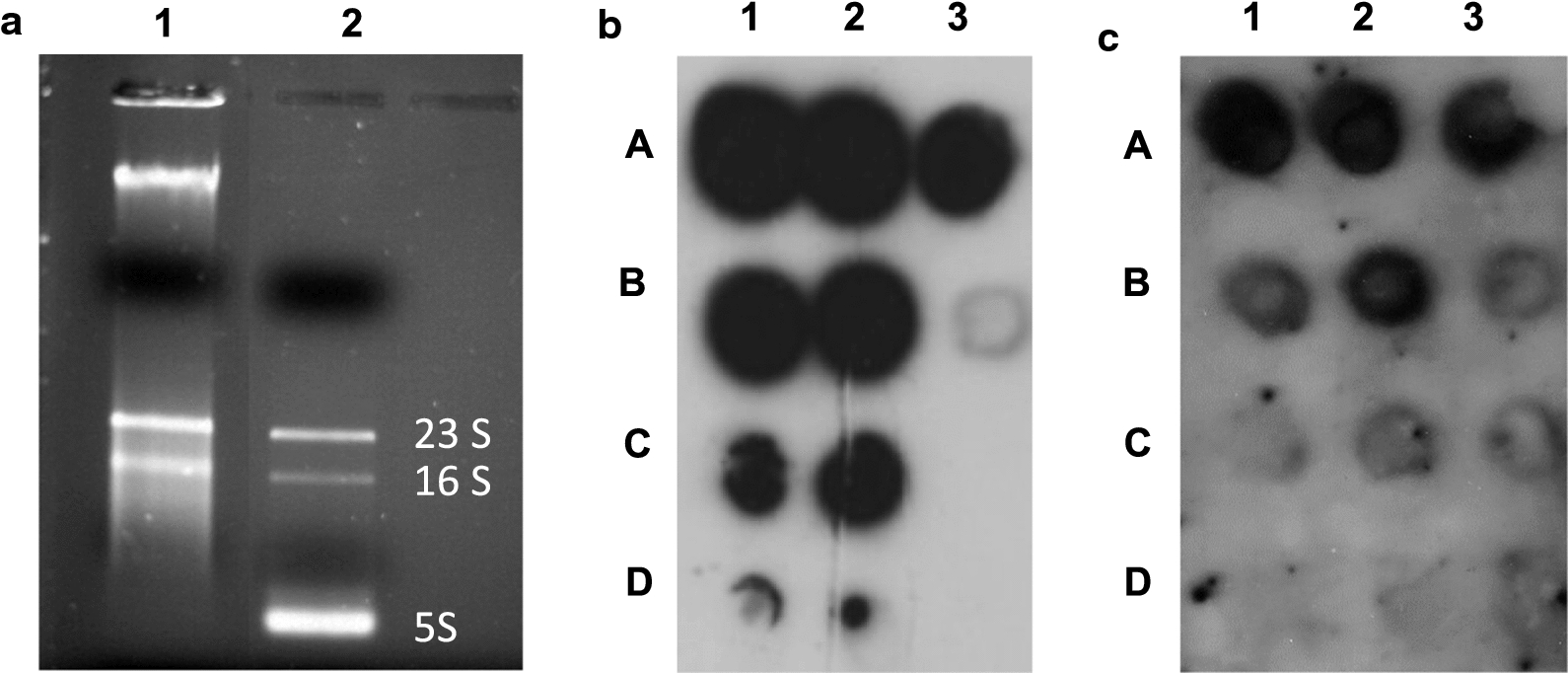
**a** Agarose gel-electrophoresis of RNA, isolated from purified hIFNγ IBs. *1*—RNA, isolated from purified hIFNγ IBs; *2*—total RNA, isolated from non-transformed *E. coli* cells. **b**, **c** Dot-blot hybridization of RNA isolated from hIFNγ IBs. **b** RNA was dotted on nitrocellulose filters, hybridized with ^32^P-labelled oligonucleotide specific for hIFNγ mRNA, striped and **c** reprobed with oligonucleotide specific for the *E. coli* 16S rRNA. Total amount of RNA on the dots: *A1* and *A2*—10 µg; *B1* and *B2*—5 µg; *C1* and *C2*—2.5 µg; *D1* and *D2*—1 µg; *A3*—10 µg RNA pre-treated with DNase; *B3*—10 µg total RNA isolated from transformed *E. coli* cells; *C3* and *D3*—10 µg total RNA isolated from non-transformed *E. coli* cells

To characterize the RNA in highly purified IBs, RNA was isolated using TRIzol^®^ under conditions recommended by the producer [42]. The obtained UV spectrum was typical for RNA and showed absorption maxima at 260 nm (Additional file 1: Fig. S2). The amount of the total RNA related to that of the IBs showed that its average content was 0.2 mg per 1 g wet purified IBs. The electrophoretic pattern of the obtained sample (Fig. 4a) showed that it consists of three main fractions—a slow-mobility one (high molecular mass), similar to the one isolated by the phenol–chloroform extraction (Fig. 3a) and two others corresponding to the bacterial 23S and 16S rRNA. The latter means that whole ribosomes have been entrapped at the time of recombinant hIFNγ aggregation and IBs formation.

To shed light on the origin of the RNA isolated from IBs, hybridization was carried out with two radiolabelled oligonucleotides of which one was complementary to the 5′ end of the hIFNγ mRNA and the second was complementary to the *E. coli* 16S rRNA. As shown in Fig. 4b, c, both oligonucleotides demonstrated specific hybridization with the RNA sample isolated from purified IBs. The hybridization signal in the DNase-treated sample (Fig. 4b, A3) proved that hIFNγ mRNA, but not plasmid DNA encoding the hIFNγ gene was present in the samples.

## Discussion

Inclusion bodies formation in bacterial cells overexpressing eukaryotic proteins is a well-known phenomenon. Although the IBs are routinely used for isolation of recombinant proteins, systematic studies on their chemical composition and mechanism of their formation are sporadic and rare. There is no consensus on whether the IBs formation is a specific process affecting the recombinant protein only or it happens with the participation of other cellular components. Hartley and Kane [35] have identified ribosomal RNA, RNA polymerase subunits and various forms of plasmid DNA in bovine somatotropin IBs. Since the latter have been non-purified, it is not clear whether the observed substances are genuine components of the IBs or co-precipitated cellular impurities. Rinas and Bailey [24] found ribosomal proteins L7/L12 in TEM β-lactamase and β-galactosidase IBs, considering them as co-precipitated protein contaminants. In NMR studies Wasmer and co-workers [38] found RNA in HET-s(218–289) IBs washed 3 times with pure water that has not been further observed after additional purification. Other authors have detected nucleic acids in IBs, but they have regarded them as non-specific impurities rather than as their genuine components [24, 26]. Chaturvedi and co-workers [34] observed that the δ-endotoxin CryIAc exists in *E. coli* IBs in the form of tight complexes with chromosomal and plasmid DNA. However, this might be due to the presence of a large hydrophobic domain in the molecule of the recombinant δ-endotoxin or to a poor purification of the IBs.

To minimize the impurities in IBs, we developed an elaborated procedure for purification of hIFNγ IBs by introducing a centrifugation step on sucrose cushion followed by twofold ultrasonication and extensive washing between the latter steps with non-denaturing concentrations of urea. Literature data show that sonication of IBs destroys the non-covalent interactions between proteins and nucleic acids thus helping their separation by centrifugation [43]. Based on this study we believe that the extensive ultrasonication followed by washing is critical for the efficient removal of co-precipitated nucleic acids from the IBs. The bacterial growth test (see Fig. 1) shows that these additional steps improve also the disintegration of the residual bacterial cells in the IBs fraction. Our results are supported also by the results of Futami and co-authors [44] who extracted equal amounts of nucleic acids from extensively sonicated IBs and IBs treated with a mixture of DNase and RNase. Thus we assume that the nucleic acids found in highly purified hIFNγ IBs are their genuine components.

As mentioned above, exploring several recombinant proteins Futami and co-authors [44] have obtained DNA and RNA from IBs treated with nucleases and concluded that the inclusion bodies contain nucleic acids that are tightly bound to the expressed unfolded protein. They have suggested that the most important factor causing aggregates during the folding is the electrostatic interaction between unfolded protein and anionic contaminants such as nucleic acids. Our results support this conclusion. Furthermore, we characterized the nature of the RNA obtained from hIFNγ IBs as hIFNγ mRNA and *E. coli* ribosomal RNA. We explain this by the fact that the processes of replication, transcription and translation are not space and time segregated in bacteria, which allows immediate interactions between the expression plasmid DNA, mRNA, translating ribosomes and newly synthesized polypeptide chains (both completely and partially folded). This is particularly valid for the expression of genes under strong constitutive promoters and strong SD sequences, as is the case with the expression plasmid pP1SD-hIFNγ used in this study. Our assumption is supported by the fact that the initiation of the chromosomal DNA replication in *E. coli* is dependent on its transcriptional activity [45] where a direct interaction between DnaA (a bacterial replication initiator protein) and RNA polymerase has been found. In addition, we have previously shown that the segregation of the expression plasmid pP1SD-hIFNγ strongly depends on the level of hFNγ-mRNA in *E. coli* cells [46, 47].

Probably many factors, such as amino acid composition, solubility, affinity to the cell membrane and other properties of the recombinant protein might affect the formation of inclusion bodies and the captivation of nucleic acids and even whole ribosomes. We speculate however, that the natural platform for this phenomenon is the lack of compartmentalization in bacteria due to which the genetic processes occur in one “test tube” contributing to the electrostatic interaction between unfolded protein and nucleic acids. Therefore, we assume that nucleic acids might be components of IBs formed by different target proteins. The aggregation of proteins (including recombinant hIFNγ) in *E. coli* cells and therefore the IBs formation could be further enhanced by the non-enzymatic glycosylation (glycation) that occurs in vivo [48–50]. The advanced glycation end products (AGEs) promote protein–protein and protein-nucleic acids cross-linking [51] that might contribute to an even more complex structure of the *E. coli* IBs.

## Conclusions

Based on the results presented in this study we conclude that the highly purified IBs isolated from *E. coli* cells overexpressing recombinant hIFNγ, contain tightly bound nucleic acids, which might be regarded as an integral part of their structure. They cannot be removed by extensive sonication and washing with non-denaturing urea solutions. They represent both sheared DNA and RNA that is composed of ribosomal and target protein mRNA. Although the role of nucleic acids in the formation of IBs remains to be clarified, we suppose that they come from gene expressing complexes (expression plasmid, mRNA and translating ribosomes) and actively participate in the aggregation process by electrostatic interactions with unfolded or partially folded proteins. Further experiments are needed to show whether these finding are valid for other recombinant proteins aggregating in IBs. Besides its fundamental significance, this finding is important for the biotechnological practice. It could serve as basis for development of new technologies for manufacturing of recombinant proteins based on preliminary removal or suppression of the co-aggregation of nucleic acids in IBs. The proteins thus obtain is expected to have improved purity and stability in water solutions.

## Methods

### Isolation of crude hIFNγ IBs fraction

*Escherichia coli LE391* cells were transformed with a plasmid for constitutive expression of hIFNγ. The cells were grown and lysed in 200 ml of 1 M urea, 0.4 M guanidinium hydrochloride, 20 mM Tris–HCl, pH 8.8 by ultrasonic disintegration as described by Petrov et al. [41]. The pellet was collected by centrifugation at 14,000 rpm for 30 min and stored at 4 °C.

### Purification of hIFNγ IBs

The crude pellet of hIFNγ IBs was suspended in TE buffer pH 7.4, sonicated (3 cycles of 1 min at amplitude 50%) and 100 μg/ml lysozyme was added at a 1/10 v/v ratio. The suspension was incubated at 37 °C for 1 h, overlaid on a 20% sucrose cushion at 1/3 v/v ratio and centrifuged at 4 °C for 20 min at 5000 rpm. The cell pellet was removed and the supernatant above the sucrose layer was collected and centrifuged at 4 °C for 15 min at 13,000 rpm. The pellet was suspended in washing buffer (0.02 M Tris, pH 8,8; 1 M urea and 0.01 M EDTA) at 1/10 v/v ratio, homogenized on a vortex mixer and centrifuged at 4 °C for 15 min at 14,000 rpm. After centrifugation, the supernatant was discarded and the pellet was re-suspended again in washing buffer. After sonication (3 cycles of 1 min at amplitude 50%) and centrifugation under the same conditions, the pellet was suspended in 1/10 v/v washing buffer.

### Analysis of the IBs for presence of viable bacterial cells

The presence of viable bacterial cells was monitored by seeding on a solid LB agar (1.7%), supplemented with tetracycline (12.5 mg/ml) and ampicillin (100 mg/ml), at each purification step.

### Analysis of the IBs protein composition

The protein pattern of the IBs fraction was monitored by Laemmli SDS-polyacrylamide gel electrophoresis. Prior loading on the 15% SDS-polyacrylamide gel, the IBs sample was resuspended in loading buffer (4× buffer: 400 mg SDS, 5 ml 0.5 M Tris pH, 6.8, 5 ml 100% glycerol, bromo-phenol blue), incubated for 5 min at 95 °C, and centrifuged for 5 min at 12,000 rpm.

### Isolation of nucleic acids from IBs

Purified IBs were suspended in 10 v/v washing buffer, incubated at 37 °C for 15 min followed by 5 min at 60 °C and cooled on ice. The isolation of nucleic acids with phenol–chloroform was carried out according to Sambrook and Russell [52]. The obtained nucleic acids were precipitated by 1/3 v/v ethanol at − 20 °C [53]. The pellet was collected by centrifugation and dissolved in 50 μl TE-buffer (pH 7.4). Nucleic acids concentration was measured by Nanodrop^®^ ND-1000 (NanoDrop Technologies, Inc., USA) at λ = 260 nm and the electrophoretic pattern was determined by standard agarose gel electrophoresis.

### Enzymatic treatment of nucleic acids isolated from IBs

Nucleic acids extracted from purified IBs were treated with RNAse A (Thermo Scientific™, 10 mg/ml), RNase T1 (Thermo Scientific™, 1000 U/ml), DNAse I, restriction endonucleases XhoI and HindIII (New England BioLabs) and Proteinase K (Roche, 10 μg/ml) following the manufacturers’ protocols.

### Isolation of RNA from hIFNγ IBs

RNA was extracted from purified IBs by TRIzol^®^ reagent (Invitrogen™) following the manufacturer’s protocol. After the last step, RNA was dissolved in 50 μl DEPC treated H_2_O (AppliChem) and stored at − 70 °C.

### Determination of hIFNγ-mRNA and *E. coli* 16S RNA in purified IBs

hIFNγ-mRNA and *E. coli* 16S RNA were determined by hybridization with 19 nt and 20 nt long ^32^P-labelled oligonucleotides specific for hIFNγ gene and *E. coli* 16S rRNA respectively as previously described [54].

## Supplementary information

**Additional file 1: Fig. S1.** UV spectra of nucleic acids isolated from purified IBs by phenol–chloroform extraction and precipitation with ethanol. The probe was analysed by NanoDrop^®^. **Fig. S2.** UV spectra of RNA isolated from purified IBs by TRIzol^®^ and analysed by NanoDrop^®^.

## Data Availability

All data generated or analyzed during this study are included in this article.

## Abbreviations

IBs: Inclusion bodies
hIFNγ: Human interferon-gamma
PAAG electrophoresis: Polyacrylamide gel electrophoresis
AGEs: Advanced glycation end products
LB: Luria-Bertani (LB) broth
DEPC: Diethylpyrocarbonate
