# Supplementary material for: Nucleic acids in inclusion bodies obtained from E. coli cells expressing human interferon-gamma
Source: Microb Cell Fact. 2020 Jul 11;19:139. doi: 10.1186/s12934-020-01400-6 (PMC7353671; doi:10.1186/s12934-020-01400-6)
Supplement: Supplementary file 1 — Additional file 1: Fig. S1. UV spectra of nucleic acids isolated from purified IBs by phenol–chloroform extraction and precipitation with ethanol. The probe was analysed by NanoDrop®. Fig. S2. UV spectra of RNA isolated from purified IBs by TRIzol® and analysed by NanoDrop®. [file 12934_2020_1400_MOESM1_ESM.docx]

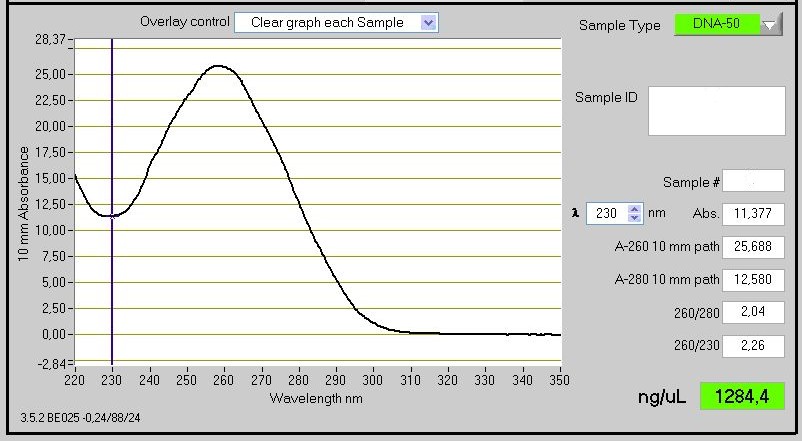


**Fig. S1: UV spectra of nucleic acids isolated from purified IBs by phenol-chloroform extraction and precipitation with ethanol.** The probe was analysed by NanoDrop®.


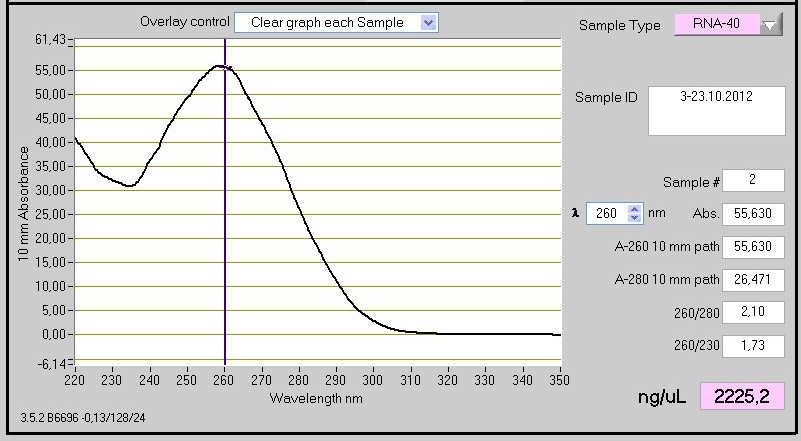


**Fig. S2: UV spectra of RNA isolated from purified IBs by TRIzol® and analysed by NanoDrop®.**
